# Draft Genome Sequences of 6 Actinobacterial Strains Isolated from Rock Surfaces Obtained from Indian Stone Ruins in Tamil Nadu, India, and Rocks from New England, United States

**DOI:** 10.1128/mra.00024-22

**Published:** 2022-02-24

**Authors:** Nathaniel J. Ennis, Dhanasekaran Dharumadurai, Louis S. Tisa

**Affiliations:** a Department of Molecular, Cellular and Biomedical Sciences, University of New Hampshire, Durham, New Hampshire, USA; b Department of Microbiology, Bharathidasan University, Tiruchirappalli, Tamil Nadu, India; University of Maryland School of Medicine

## Abstract

Here, we report the draft genome sequences obtained for 6 actinobacterial strains isolated from stone surfaces acquired from New England and Indian ruins. These strains were sequenced to determine their potential functional roles in the stone microbiome. The strains belong to the genera *Allobranchiibius*, *Agrococcus*, *Dermococcus*, *Leifsonia*, and Mycobacterium.

## ANNOUNCEMENT

Stone surfaces are extreme environments that support microbial life. We have been investigating the effects of stone lithology and environmental conditions on stone microbiomes from several different regions, including North Africa, India, and New England, United States (1–3). Besides conducting culture-independent studies, we have also isolated bacterial strains from these stone samples (3).

Stone samples from India (1) and New England (3) were collected aseptically using a sterilized rock hammer or chisel. The samples were crushed aseptically with a sterile rock hammer and further reduced to a powder by grinding with a sterile mortar and pestle. The pulverized stone was serially diluted in phosphate-buffered saline and plated onto Czapeck (4), R2A (5), Luedemann (6), and starch casein (4) medium containing cycloheximide. About 74 isolates were obtained initially, purified, and propagated on Czapeck, R2A, Luedemann, or starch casein medium. These isolates were incubated in Czapeck broth medium for 3 to 5 days at 28°C, and genomic DNA (gDNA) was extracted by the cetyltrimethylammonium bromide (CTAB) DNA extraction protocol (7). RNA was removed by RNase treatment. The quality and quantity of the gDNA were verified using a Thermo Scientific Nanodrop instrument. The isolates were identified initially by amplifying and sequencing their 16S rRNA genes. Based on these results, 6 isolates were chosen for whole-genome sequencing analysis to provide insight into rock microbiome function (Table 1).

**TABLE 1:** Genome statistics

Bacterial species	Isolate^*a*^	Accession no.	SRA accession no.	No. of reads	No. of contigs	Avg coverage (×)	Genome assembly size (bp)	*N*_50_ contig size (bp)	No. of CDSs^*b*^	G+C content (%)	No. of rRNAs	No. of tRNAs
*Leifsonia* sp.	TF02-11	JAGEKP000000000	SRR13614795	12,933,062	50	627	5,087,634	293,374	4.918	69.5	3	48
*Dermacoccus* sp.	NHGro5	JAGEKQ000000000	SRR13747839	11,872,410	66	942	3,103,234	112,683	2,783	69.0	8	53
*Agrococcus* sp.	TF02-05	JAGEKO000000000	SRR13615610	12,483,940	16	1,142	2,666,021	307,215	2,612	71.9	3	45
*Allobranchiibius* sp.	CTAmp26	JAGEKR000000000	SRR13754888	9,844,656	33	653	3,713,509	197,609	3,490	69.5	5	43
*Allobranchiibius* sp.	GilTou38	JAGEKN000000000	SRR13615609	13,649,130	18	936	3,588,242	335,228	3,397	69.7	3	46
Mycobacterium sp.	PS03-16	SPQO00000000	SRR8784755	3,589,924	91	222	5,507,058	140,871	5,314	69.9	5	46

a Bacteria were isolated on Czapeck (TF02-05, CTAmp26, and PS03-16), R2A (TF02-11 and GilTou38), and Luedemann (NHGro5) media. All bacterial strains were grown on Czapeck medium for 3 to 5 days before gDNA isolation. Stone sample location and lithology are listed as follows: TF02, granite from outside rock damage area at Fort Tiruchirappalli, Tamil Nadu, India; PS03, granodiorite from the inside temple wall damage area at Valikandapuram Sivan Temple, Tamil Nadu, India,; NHGro, granite found on the grounds near pulp mill at Livermore Hollow, Holderness, NH; and CTAmp, amphibolite found in surrounding area by mill site at Gay City State Park, Hebron, CT.

b CDSs, coding DNA sequences.

Whole-genome sequencing was performed at the Hubbard Center for Genome Studies (University of New Hampshire, Durham, NH) using Illumina technology techniques (8). A paired-end library was constructed using a Nextera DNA library preparation kit (Illumina, San Diego, CA) and sequenced on an Illumina HiSeq 2500 instrument to produce 250-bp paired-end reads. Total numbers of reads for all 6 strains are listed in Table 1. The Illumina sequence data, except for PS03-16, were trimmed and assembled using CLC Genome Workbench *de novo* assembly version 21.0.1 using default parameters. The Illumina sequence data from PS03-16 were trimmed by Trimmomatic version 0.36 (9) and assembled using SPAdes version 3.13 (10). Leading and trailing bases below a quality of three were trimmed. The reads were then scanned with a sliding window of 4 bp and trimmed if the average quality dropped below 30. Finally, reads were dropped if the length was less than 25 bp. The assembled genomes were annotated via the NCBI Prokaryotic Genome Annotation Pipeline (PGAP) (11). The assembly metrics and annotation features are given in Table 1. The identities of the strains were determined by a whole genome-based taxonomic analysis via the Type (Strain) Genome Server (TYGS) platform (12) (https://tygs.dsmz.de) including digital DNA:DNA hybridization (dDDH) values (13). The type-based species clustering using a 70% dDDH radius around each of the type strains was used as described previously (14), while subspecies clustering was done using a 79% dDDH threshold as introduced previously (15). Among the six strains, three potential new species of the genera *Allobranchiibius*, *Leifsonia*, and Mycobacterium were identified. A bioinformatic analysis of these genomes by the use of the antiSMASH program (16, 17) revealed the presence of secondary metabolic biosynthetic gene clusters. Many of these potential natural products should be involved in the rock-microbe interactions and aid in their community structure.

### Data availability.

The draft genome sequences of these bacterial strains have been deposited in GenBank under the accession numbers listed in Table 1. Both the assembly and raw reads are available at DDBJ/ENA/GenBank under BioProject numbers PRJNA694661 and PRJNA480027.
